# Editorial: Normalization of human social and emotional dysfunction using noninvasive brain modulation approaches: From proof-of-concept to clinical studies

**DOI:** 10.3389/fnhum.2023.1142894

**Published:** 2023-01-24

**Authors:** Shuxia Yao, Jana Zweerings, Xiaochu Zhang, Klaus Mathiak

**Affiliations:** ^1^The Clinical Hospital of Chengdu Brain Science Institute, School of Life Science and Technology, University of Electronic Science and Technology of China, Chengdu, Sichuan, China; ^2^Department of Psychiatry, Psychotherapy and Psychosomatics, Faculty of Medicine, RWTH Aachen, Aachen, Germany; ^3^Department of Psychology, School of Humanities and Social Science, University of Science and Technology of China, Hefei, Anhui, China; ^4^Department of Radiology, The First Affiliated Hospital of USTC, Hefei National Research Center for Physical Sciences at the Microscale and School of Life Science, Division of Life Science and Medicine, University of Science and Technology of China, Hefei, China; ^5^Application Technology Center of Physical Therapy to Brain Disorders, Institute of Advanced Technology, University of Science and Technology of China, Hefei, China

**Keywords:** mental disorders, social and emotional dysfunction, brain modulation, innovative, translational potential

Mental disorders, such as depression, anxiety, and autism, have become one of the leading causes of disability worldwide with a high lifetime prevalence. Individuals with these disorders exhibit marked social and cognitive impairments, which are often closely associated with brain alterations both structurally and functionally. However, success rates of pharmacological and behavioral interventions vary widely and affected individuals often suffer from residual symptoms. Further, some of these interventions are characterized by severe side effects and induce high drop-out rates. Therefore, innovative brain modulation approaches that can directly target and regulate the involved brain circuits are of great translational potential for these disorders.

Our Research Topic aimed to improve our understanding of how innovative brain modulation approaches affect human behavior and neural responses and provide an overview on recent progress in the field. The articles that were published in this Research Topic bring us new insight on how brain modulation approaches such as real-time functional magnetic resonance imaging (rt-fMRI) neurofeedback (NF) training, transcranial magnetic stimulation (TMS), and transcranial direct current stimulation (tDCS) can be applied to modify emotion regulation and brain activity/connectivity in healthy or clinical populations. More specifically, in a proof-of-concept study on emotion regulation Kerr et al. explored an innovative dyadic rt-fMRI NF protocol in which mothers attempted to down-regulate the anterior insula activity of their children. Based on an independent support vector machine-based classifier, Pereira et al. trained patients with depression to match their own neural activity with the neural activity corresponding to the “happiness emotional brain state” of a healthy participant *via* rt-fMRI NF training and found improvement in clinical symptoms associated with training success. Orth et al. conducted a systematic review of rt-fMRI NF studies targeting brain activations within the frontostriatal circuitry given that it is a common target for the treatment of aberrant behaviors in various psychiatric and neurological disorders. Their overview emphasizes the relevance to address network connectivity rather than localized excitability only.

Using the online single-pulse TMS technique, Cheng et al. investigated the asymmetric role of the bilateral ventrolateral prefrontal cortex (VLPFC) in cognitive reappraisal and found that while the left VLPFC is responsible for the semantic process of generating and selecting appraisal strategies based on the goal of emotion regulation, the right VLPFC is mainly involved in inhibiting inappropriate negative emotions and thoughts. Huang et al. demonstrated that a 4-week intervention of high-frequency repeated TMS on the left dorsolateral prefrontal cortex (lDLPFC) decreased the temporal variability of resting-state functional connectivity (rsFC) in the cortico-thalamo-cerebellar circuit (CTCC), with higher alteration in rsFC temporal variability between the left DLPFC and right posterior parietal thalamus predicting a higher remission ratio of negative symptom severity. Importantly, in the support vector regression analysis the pattern of rsFC temporary variability within the CTCC predicted the efficacy of high-frequency rTMS intervention on negative symptoms of schizophrenia. By combining tDCS with TMS, Alkhasli et al. revealed that excitatory intermittent theta-burst (iTBS) to a tDCS-inhibited lDLPFC yielded more robust functional connectivity to various areas as compared to excitatory iTBS to a tDCS-enhanced DLPFC. This suggests feasibility of using tDCS to modulate subsequent TMS effects on the DLPFC which may be of translational potential for the treatment of depression. In addition to these direct brain modulation approaches, Wang et al. demonstrated that open motor skill training (table tennis experts vs. matched non-experts) may be associated with greater activation in brain regions involved in visual processing during language processing tasks (word and semantic judgement tasks). These findings bridge the complex interplay between short- and long-term neuromodulation and behavioral training effects.

In conclusion, studies in the Research Topic applied highly innovative approaches to modulate human brain functions in healthy or clinical populations, with some of them reporting promising improvement in clinical symptom severity. We believe findings from these studies does not only improve our understanding of neural mechanisms underlying these brain modulation approaches, but also inspire future studies aiming to translate neuromodulatory approaches from different disciplines into clinical applications.

## Author contributions

SY and KM drafted the manuscript. JZ and XZ critically revised the manuscript. All authors contributed to the article and approved the submitted version.

